# Optimizing blood management in arthroplasty: a meta-analysis of carbazochrome sodium sulfonate and Tranexamic acid combination

**DOI:** 10.1186/s13018-025-06038-x

**Published:** 2025-07-17

**Authors:** Mohamed A. Alsaied, Omnia Samy El-Sayed, Shahd Alqato, Abdelrahman M. Elettreby, Ahmed A. Abo Elnaga

**Affiliations:** 1 Faculty of Medicine, Mansoura University, Mansoura, Egypt; 2 Faculty of Medicine, Zagazig University, Zagazig, Sharqia Egypt; 3 Internal Medicine Department, Arab Medical Center, JOR Amman, Jordan

**Keywords:** Arthroplasty, Blood transfusion, Carbazochrome sodium sulfonate, Tranexamic acid

## Abstract

**Background:**

Persistent bleeding and inflammation during and after surgery are frequent problems in hip and knee surgeries that lead to high blood transfusion needs and slow recovery. Tranexamic acid (TXA) is a popular agent used to control bleeding, but its efficacy may be improved when combined with Carbazochrome sodium sulfate (CSS), capillary hemostatic agent that stabilizes microvascular integrity and reduces capillary bleeding. This meta-analysis compares the efficacy and safety of CSS plus TXA and TXA alone in controlling bleeding during surgery, inflammation, and postoperative outcomes.

**Methods:**

A thorough literature search was performed across multiple databases until January 2025 to identify pertinent randomized controlled trials comparing the efficacy and safety of the combination of Carbazochrome sodium sulfate and Tranexamic acid against Tranexamic acid alone for the reduction of blood loss. The study’s primary outcomes were total blood loss, hidden blood loss, intraoperative blood loss, and maintenance of hemoglobin levels. The quality of the studies included was evaluated utilizing the RoB 2 tool. Subsequent to data extraction, a meta-analysis was conducted utilizing RevMan 5 software with a random effects model.

**Results:**

This systematic review identified six studies (*n* = 800 patients) fulfilling research criteria. The meta-analysis has shown that there was a robust reduction in total blood loss (MD = -230.92 mL, 95% CI [-271.69 to -190.14], *P* < 0.00001) and hidden blood loss (MD = -220.52 mL, 95% CI [-263.78 to -177.27], *P* < 0.00001) when comparing the intervention group with TXA alone with topical administration providing less blood loss than Intravenous. In addition to the above measure, hemoglobin preservation was also improved (MD = -0.59 g/dL, 95% CI [-0.73 to -0.46], *P* < 0.00001). Furthermore, compared to TXA alone, the combination group had much lower requirements for blood transfusion (RR = 0.13, 95% CI [0.04 to 0.38], *P* = 0.0003) while there was no increase in complication with wounds or venous thromboembolism.

**Conclusion:**

The use of carbazochrome sodium sulfonate (CSS) combined with tranexamic acid (TXA) proves to be more effective at controlling bleeding, hemoglobin loss, postoperative inflammation, and pain after hip and knee arthroplasty than using TXA alone. There was also increased efficacy from topical application, as well as increased safety and decreased transfusion use. Combination therapy had good results; however, its relative inefficacy on operative duration and duration of hospital admissions indicates that more work needs to be done on this issue.

**Supplementary Information:**

The online version contains supplementary material available at 10.1186/s13018-025-06038-x.

## Introduction

Osteoarthritis (OA) is the most common non-inflammatory arthritis and joint degenerative disease (JDD) within patients over 60 years old, affecting 10% of men and 18% of women in this population worldwide [1, 2]. Among its risk factors, age is considered the most substantial, possibly combining multiple mechanisms such as increased oxidative stress, massive muscle weakening, and cartilage destruction [3]. The majority of OA affects the knee and hip joints and has been correlated to excessive joint load and injury [4, 5]. This debilitating disease, which primarily causes pain, stiffness, and limitation of movement, can dramatically affect the patient’s quality of life; however, there are some current treatment options that have been proven to be quite effective [4].

Several treatment options are available for managing degenerative joint disorders. For instance, physical therapy (PT) can reduce pain and improve joint function; however, it shows slow progression [6, 7]. On the other hand, medications such as non-steroidal anti-inflammatory drugs (NSAIDs) demonstrate faster pain relief; nonetheless, they carry a high risk of gastrointestinal and cardiovascular disorders [8, 9]. In addition, injections of corticosteroids and hyaluronic acid initially improve pain, but they can cause joint damage if used for a long time [10, 11]. Moreover, arthroscopy, a minimally invasive procedure, can relieve symptoms significantly, but it is not suitable for advanced OA [12]. In cases of severe pain and advanced JDD, not responding to conservative or medical treatments, total joint arthroplasty (TJA) is the choice. TJA encompasses various types of joint replacement surgeries, including total hip arthroplasty (THA) and total knee arthroplasty (TKA) [13, 14]. In these procedures, the damaged cartilage and bone are removed and replaced with metal, plastic, or ceramic prosthetic components designed to mimic the natural movement of healthy joints [14].

Research has shown that TJA procedures can lead to substantial improvements in pain relief, mobility, and overall quality of life. For total hip replacement, the 10-year implant survival rate was evident to be 95.6% while for total knee replacement, the 10-year implant survival rate was 96.1% [15]. To decrease blood loss during these surgical procedures, many solutions have been introduced, ranging from tourniquets to electrocautery, and recently, the use of antifibrinolytics [16]. Topical, oral, and intravenous tranexamic acid, as well as antifibrinolytic, have shown similar efficacy in reducing blood loss and the need for postoperative blood transfusion in patients undergoing THA or TKA [16–18]. A study in elderly hip fracture patients found that the implementation of a dedicated orthogeriatrician significantly improved hemoglobin levels at discharge and reduced transfusion rates, suggesting that structured perioperative care can enhance outcomes beyond pharmacological measures [19]. Finally, a narrative review highlighted that multifaceted strategies—incorporating preoperative anemia management, optimized analgesia, drainage techniques, and perioperative anticoagulation—play a pivotal role in reducing transfusion needs [20].

Carbazochrome sodium sulfonate (CSS) is an anti-hemorrhage agent which is a product of oxidation of epinephrine [21]. It helps to keep capillaries stable and promotes blood clotting [21]. However, the actual mechanism through which it works still unclear but there is some evidence that CSS reinforces microvascular integrity by enhancing the contraction of damaged capillaries as well as reducing inflammation-induced endothelial hyperpermeability [22]. Clinically, this drug has been used in various perioperative conditions. In arthroscopic rotator cuff repair, intravenous CSS improved surgical field visibility by reducing bleeding and was associated with less early postoperative pain and swelling [23]. Apart from orthopedics, CSS has also found use in urologic and otolaryngologic practice for controlling small vessel bleeding and inflammation [24, 25] thereby showing its broad range of potential applications in post-operative care.

In contrast to TXA, mixed evidence has emerged about the effectiveness of the combination of Carbazochrome Sodium Sulfonate (CSS) with TXA during surgeries in terms of blood loss reduction, RBCs transfusion, mortality, hospital stay, and complications [25–27]. Blood loss reduction is essential as an audit was performed and showed that many transfusions in orthopedic surgery appear to be based on routine rather than clinical need [28]. Yet, there is not enough evidence of options to optimally reduce blood loss during these procedures. This systematic review and meta-analysis aim to evaluate the efficacy of combined CSS and TXA therapy compared to TXA alone in patients undergoing TKA or THA. The primary outcomes assessed include blood loss, postoperative inflammation, and pain control, with the goal of providing clearer clinical guidance on the use of CSS in joint arthroplasty.

## Methods

### Protocol and registration

Prior to start of this systematic review, a predesigned established methods and fine criteria were made, and protocol of this study was prospectively registered at PROSPERO (CRD42025641159). This systematic review and meta-analysis were conducted according to instructions stated in Cochrane handbook of systematic review and meta-analysis (version 5.1.0) [29] and was written in coherence with guidelines of PRISMA statement [30].

### Eligibility criteria

#### Inclusion criteria

To ensure inclusion or relevant studies to our question, we included publications in English that fall under these PICOS specifications:

- *Population*: Patients undergoing Total hip arthroplasty (THA) or total knee arthroplasty (TKA), either unilateral or simultaneous bilateral procedures.
- *Intervention*: Intravenous (IV) Tranexamic acid in combination with Carbazochrome Sodium Sulfonate (CSS), either topical -directly into or around the joint- or IV.
- *Comparator*: IV Tranexamic acid alone combined with placebo.
- *Outcomes*: Studies that evaluate outcomes related to operative bleeding, as well as postoperative performance metrics, including:

***i.*Primary outcomes**:

- Total blood loss.
- Intraoperative blood loss.
- Hidden blood loss.
- Hemoglobin reduction.

***ii.*Secondary outcomes**:

- Post operative pain (at day one, two and three PO).
- Inflammatory markers including CRP, ESR and IL-6 (at day one, two and three PO).
- Operation time.
- Hospital stay time.

***iii.*Safety outcomes**:

Any reported post operative complications including need for blood transfusion, IM venous thrombosis or wound complications.

- Study designs: Randomized controlled trials will only be included.

#### Exclusion criteria

Studies that met any of the following criteria were excluded: (1) non-peer-reviewed studies or study designs other than randomized controlled trials (e.g. narrative reviews, commentaries, editorials, observational studies, case reports, and conference abstracts), (2) studies including population other than identified in inclusion criteria (e.g. animal studies, non-surgical cases or surgeries other than THA or TKA), (3) studies including other interventions or non-adequate comparators, (4) studies non-focusing on operative blood loss, (5) studies published in languages other than English.

### Information sources and search strategy

Database searching of pertinent studies was carried out across PubMed, Cochrane Central Register of Controlled trials, Scopus, Web of Science and clinical registry databases from inception till 5th January 2025 using the following key words: (Carbazochrome Sodium Sulfonate), (Tranexamic Acid), (Hip Arthroplasty), (knee arthroplasty) and (Joint Replacement Surgery). No restrictions or limitations of search were made ensuring inclusion of all potentially relevant studies.

### Study selection and data extraction

#### Selection process

At first, records of databases search were imported into Endnote [31] to detect and remove duplicates, then the remaining records were imported to Rayyan website [32] for screening steps. Initially, titles and abstracts were screened for eligibility then a thorough examination of full texts was conducted to test potential studies against our criteria. Screening steps were done by two independent reviewers and any discrepancies between them was solved by third reviewer opinion.

#### Data extraction

Two independent authors carried out data extraction manually using an online data extraction form (Google Sheets). Detailed information was carefully extracted from all eligible trials-including study ID (first author’s name + year of publication), details of intervention and comparator, type of surgery, post operative follow up period. Moreover, patients’ demographics and baseline characteristics were extracted including age, BMI, preoperative blood indices, ASA status and operative time were extracted.

### Assessment of risk of bias

Assessment of individual risk of bias of each study was conducted using Version 2 of the Cochrane risk-of-bias tool for randomized trials (RoB 2) [33]. RoB2 encompasses seven domains: randomization processes, deviations from the intended intervention, missing outcome data, outcome measurement, and selection of reported results. Each domain was judged as “low risk”, “some concerns” or “high risk”. Assessment of risk of bias was conducted independently by two investigators and any disagreement between them was solved by discussion and third reviewer opinion if needed.

### Statistical analysis and heterogeneity

Meta-analysis was performed using Review Manager software (RevMan v.5.4) [34] on the extracted outcome data that present in at least 3 of the included studies. Regarding continuous outcomes, data was pooled as mean difference (MD) with 95% confidence interval (CI). For binary outcome data, the number of events and the total patient count were pooled to determine the risk ratio (RR) along with a 95% confidence interval (CI). The level of statistical significance was set to be *p* < 0.05. If SD deviation was missing, it was calculated using other statistical measures such as interquartile range (IQR), and CI. The change from baseline values were extracted instead of final values. In case of the absence of the SD of change, final values were extracted. Data represented by figures were extracted using Plot digitizer online app [35]. A random effect model (Inverse variance) was adopted rather than a fixed effect model yielding a more conservative estimate of the pooled effect and generalizable results. To evaluate the presence and degree of heterogeneity, we used the Chi-square and I-square tests, respectively, as outlined in chapter nine of the Cochrane Handbook 39. We interpreted the I-square test results, which indicates the variation across studies due to heterogeneity rather than chance, as follows: 0–40% were considered insignificant, 40–60% were considered moderate, and more than 60% were considered substantial%. Heterogeneity was considered significant if the alpha level for the Chi-square test was below 0.1. In cases of significant heterogeneity, we conducted a sensitivity analysis of the data or subgroup analysis. A subgroup analysis was performed based on CSS route and joint type. Finally, we could not assess publication bias using funnel plots due to the limited number of included studies. Additionally, leave-one-out sensitivity analysis was performed using R software [36] to determine the robustness of the pooled effect size meta-analysis results by systematically excluding one study and recalculating the pooled effect size. Hartung-Knapp adjustment was adopted so that all results are more robust and consider the potential unknown variation that could exist among the studies.

## Results

### Study selection

**Fig. 1:**
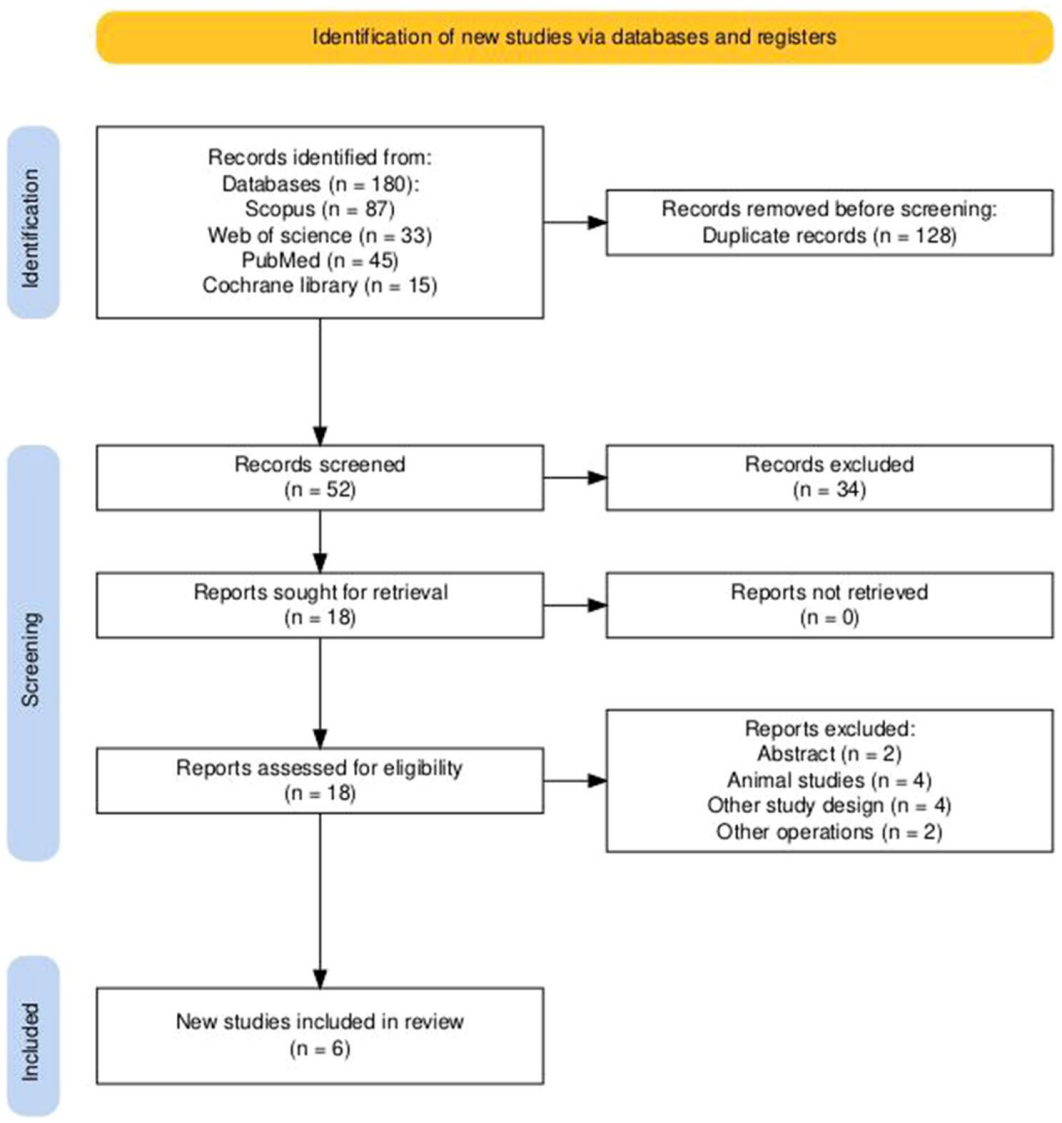
PRISMA flow diagram of the selection process

Database search process yielded identification of 180 records; 128 duplicate records were removed resulting in 52 final records that undergone screening steps. Titles and abstracts screening process of 52 records led to elimination of 34 records that didn’t meet the eligibility criteria. A total of 18 reports were retrieved and examined in full text, of which six studies [37–42] met the preset inclusion criteria and were incorporated into the systematic review and meta-analysis. Figure 1 illustrates PRISMA flow diagram of selection process.

### Characteristics of studies

This systematic review included six prospective randomized controlled trials (total sample size = 800 patients). Intervention group in all studies included Carbazochrome Sodium Sulfonate (CSS) either topical or IV in combination with 1 g IV TXA and the control group composed of IV TXA in addition to placebo. Two of the studies included in the review (Luo 2019 and Luo 2021) composed of two intervention groups (IV group and topical one). Primary total knee arthroplasty (TKA), primary unilateral total hip arthroplasty (THA), and simultaneous bilateral THA were the types of surgical procedures that were examined. Postoperative follow up ranged from 10 days up to 3 months. For each study, there were 50 participants in the experimental and control groups with mean ages varying between 53.9 and 70.5 years and a body mass index (BMI) of 23–26 kg/m². Table 1 shows detailed summary of included studies and patients’ baseline characteristics.

**Table 1:** Summary of included studies and patients’ baseline characteristics

Study ID		Onondera 2012	Luo 2019 (Topical group)	Luo 2019 (IV group)	Luo 2021 (Topical group)	Luo 2021 (IV group)	Luo 2022	Ye 2023	Han 2024
Study design		Prospective RCT	Prospective RCT		Prospective RCT		Prospective RCT	Prospective RCT	Prospective RCT
Country		Japan	China		China		China	China	China
Intervention group (I)		1 g TXA and 50 mg CSS in 50 mL saline.	1 g TXA IV 5 min before incision + 60 mL CSS in saline around and within the joint capsule before closure + 100 ml placebo 3 h. post-surgery	1 g TXA IV 5 min before incision, 60 ml placebo around joint capsule and 100 mL CSS IV 3 h. post-surgery.	1 g TXA IV just before skin incision, with 40 mg CSS before closure, and 60 ml placebo 3 h. post-surgery.	1 g TXA IV just before skin incision, with 40 ml placebo before closure, and 60 mg IV CSS 3 h. post-surgery.	1 g of TXA IV 5 min before skin incision, 40 mg CSS + 60 ml saline before closure.	1 g of TXA IV before operation, 40 mg IV CSS within 3 h after surgery.	1 g of TXA IV before surgery, 40 mg IV CSS within 3 h after surgery.
Control group (C)		50 mL of saline.	1 g TXA IV 5 min before incision, placebo in the joint capsule and 3 h. post-surgery.	1 g TXA IV 5 min before incision, placebo in the joint capsule and 3 h. post-surgery.	1 g TXA IV just before skin incision, placebo during operation and three hours postoperatively.	1 g TXA IV just before skin incision, placebo during operation and three hours postoperatively.	1 g of TXA IV 5 min before incision, 40 mg placebo + 60 ml saline before closure.	1 g of TXA IV before operation, 40 mg IV placebo within 3 h after surgery.	1 g of TXA IV before surgery, 40 mg IV placebo within 3 h after surgery.
Type of surgery		Primary total knee arthroplasty (TKA).	Unilateral primary TKA.	Unilateral primary TKA.	Unilateral primary THA.	Unilateral primary THA.	Unilateral primary THA direct anterior approach	Primary, unilateral THA direct anterior approach	SBTHA with simultaneous THA
Post-operative follow-up		10 days	3 months	3 months	3 months	3 months	2 months	3 months	3 months
# Operated side (left)	I	N/A	22 (44)	18 (36)	23 (46)	28 (56)	N/A	26 (52)	25 (50)
	C	N/A	24 (48)	24 (48)	26 (52)	26 (52)	N/A	25 (50)	22 (44)
Patients N	I	50	50	50	50	50	50	50	50
	C	50	50	50	50	50	50	50	50
Age (yrs)		70.4 ± 10.1	68.2 ± 6.6	64.5 ± 7.3	55.5 ± 12.3	57.9 ± 13	57.63 ± 12.10	53.9 ± 14.4	56.8 ± 11.6
		70.5 ± 8.3	66.7 ± 8.3	66.7 ± 8.3	58 ± 11.6	58 ± 11.6	59.40 ± 11.52	55.9 ± 11.1	57.5 ± 10.4
# Males	I	8 (16)	13 (26)	20 (40)	24 (48)	28 (56)	26 (52)	20 (40)	24 (48)
	C	9 (18)	14 (28)	14 (28)	23 (54)	23 (54)	23 (46)	22 (44)	23 (46)
BMI (kg/m^2^)	I	25.3 ± 4.6	26.12 ± 3.59	25.63 ± 3.7	23.04 ± 2.9	24.14 ± 2.41	23.04 ± 2.67	24.2 ± 3.8	23.4 ± 3.0
	C	25.9 ± 5.1	25.78 ± 3.21	25.78 ± 3.21	23.33 ± 2.99	23.33 ± 2.99	23.13 ± 2.24	23.4 ± 3.0	24.2 ± 3.8
# OA diagnosed	I	34 (68)	N/A	N/A	8 (16)	9 (18)	N/A	15 (30)	N/A
	C	39 (78)	N/A	N/A	10 ± 20	10 ± 20	N/A	12 (24)	N/A
Operation time (min)	I	117.0 ± 41.5	65.8 ± 11.2	66.6 ± 8.1	60 ± 12	60 ± 13	58.67 ± 12.31	80.1 ± 17.8	146.79 ± 30.45
	C	111.2 ± 21.3	66.5 ± 8.4	66.5 ± 8.4	62 ± 12	62 ± 12	59.47 ± 11.40	83.4 ± 20.1	145.32 ± 32.66
Transfusion rate (n)	I	N/A	N/A	N/A	0	0	N/A	0	0
	C	N/A	N/A	N/A	7	7	N/A	5	4
D-dimer (mg/l FEU)	I	1.68 ± 2.35	0.86 ± 1.03	0.69 ± 0.0.71	1.1 ± 1.4	0.8 ± 0.8	N/A	1.2 ± 1.7	1.20 ± 1.30
	C	1.97 ± 3.08	0.89 ± 0.93	0.89 ± 0.93	0.9 ± 1.0	0.9 ± 1.0	N/A	0.9 ± 1.1	1.30 ± 1.60
Hb (g/dL)	I	N/A	13.12 ± 1.45	13.64 ± 1.16	134.1 ± 15.0	140.7 ± 15.4	13.92 ± 1.66	13.5 ± 1.3	11.90 ± 1.20
	C	N/A	13.16 ± 1.5	13.16 ± 1.5	134.7 ± 14.4	134.7 ± 14.4	13.2 ± 1.35	13.6 ± 1.5	11.60 ± 1.60
Hct (L/L)	I	N/A	0.41 ± 0.04	0.42 ± 0.03	0.42 ± 0.04	0.43 ± 0.03	0.43 ± 0.05	42.0 ± 4.0	45.90 ± 3.30
	C	N/A	0.41 ± 0.04	0.41 ± 0.04	0.42 ± 0.03	0.42 ± 0.03	0.41 ± 0.03	42.0 ± 4.6	45.60 ± 3.50
INR	I	N/A	0.99 ± 0.06	1.02 ± 0.07	1.04 ± 0.2	1.06 ± 0.2	0.95 ± 0.08	1.0 ± 0.2	1.20 ± 0.11
	C	N/A	1.01 ± 0.07	1.01 ± 0.07	1.05 ± 0.2	1.05 ± 0.2	0.97 ± 0.08	1.0 ± 0.1	1.16 ± 0.20
PLT (*10^9^/L)	I	N/A	204.6 ± 76.4	221.3 ± 67.4	210.4 ± 70.7	204.9 ± 53.5	N/A	199.6 ± 78.6	201.6 ± 72.1
	C	N/A	198.1 ± 58.8	198.1 ± 58.8	196.9 ± 54.5	196.9 ± 54.5	N/A	195.5 ± 55.1	203.5 ± 75.2
# ASA I	I	N/A	7 (14)	9 (18)	7 (14)	9 (18)	5 (10)	5 (10)	0 (0)
	C	N/A	10 (20)	10 (20)	7 (14)	7 (14)	7 (14)	8 (16)	0 (0)
# ASA II	I	N/A	35 (70)	32 (64)	33 (66)	33 (66)	33 (66)	37 (74)	32 (64)
	C	N/A	31(62)	31(62)	34 (68)	34 (68)	30 (60)	34 (68)	35 (70)
# ASA III	I	N/A	8 (16)	9 (18)	10 (20)	8 (16)	12 (24)	8 (16)	18 (36)
	C	N/A	9 (18)	9 (18)	9 (18)	9 (18)	13 (26)	8 (16)	15 (30)

±: values presented as mean ± SD, #: numbers presented as N (%), RCT: randomized controlled Trial, TXA: Tranexamic Acid, CCS: Carbazochrome Sodium Sulfonate, TKA: Total Knee Arthroplasty, THA: Total Hip Arthroplasty, IV: Intravenous. SBTHA: Simultaneous Bilateral Total Hip Arthroplasty

### Risk of bias and quality assessment

**Fig. 2:**
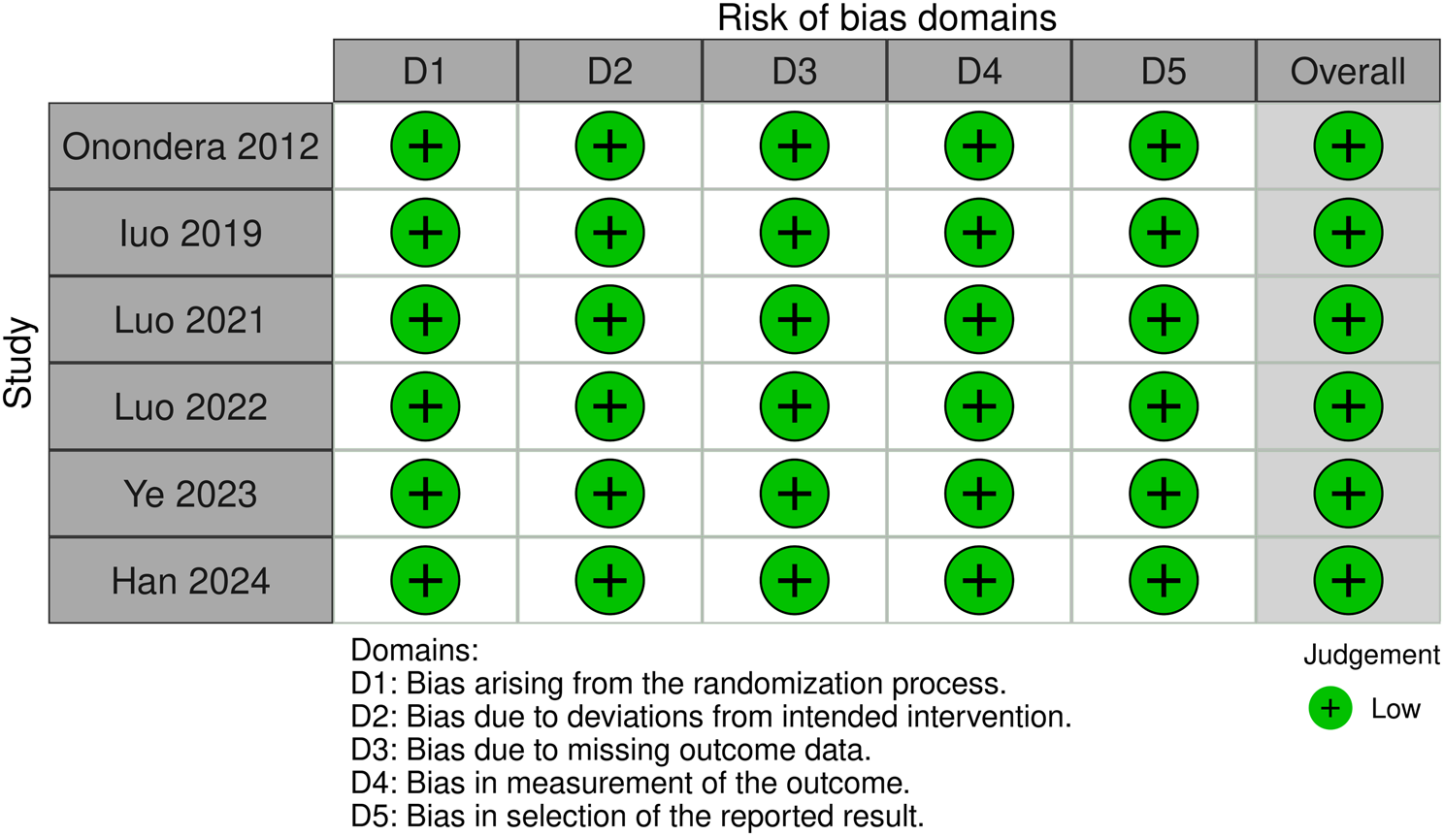
Risk of Bias assessment graph

Quality assessment of included studies was conducted using revised Cochrane risk-of-bias tool for randomized trials (RoB 2). Assessment revealed that all included studies were classified as low risk of bias across evaluated domains, supporting the reliability of the synthesized results. Figure 2 shows the risk of bias graph.

### Primary outcomes

#### Bleeding outcomes

Subgroup analyses were conducted across all bleeding outcomes, categorizing results by route of administration (intravenous vs. topical).

**1- Total blood loss (TBL)**

Meta-analysis of data from six included trials encompassing 400 patients in TXA + CSS group and 400 patients in TXA alone group was conducted. The pooled analysis demonstrated that TXA + CSS significantly reduced total blood loss compared to TXA alone (MD= -230.92 ml, 95% CI [-271.69 to -190.14]; *P* < 0.00001) with no heterogeneity detected (τ² = 0.00; Chi² = 5.38, (*P* = 0.61); I² = 0%).

**Fig. 3:**
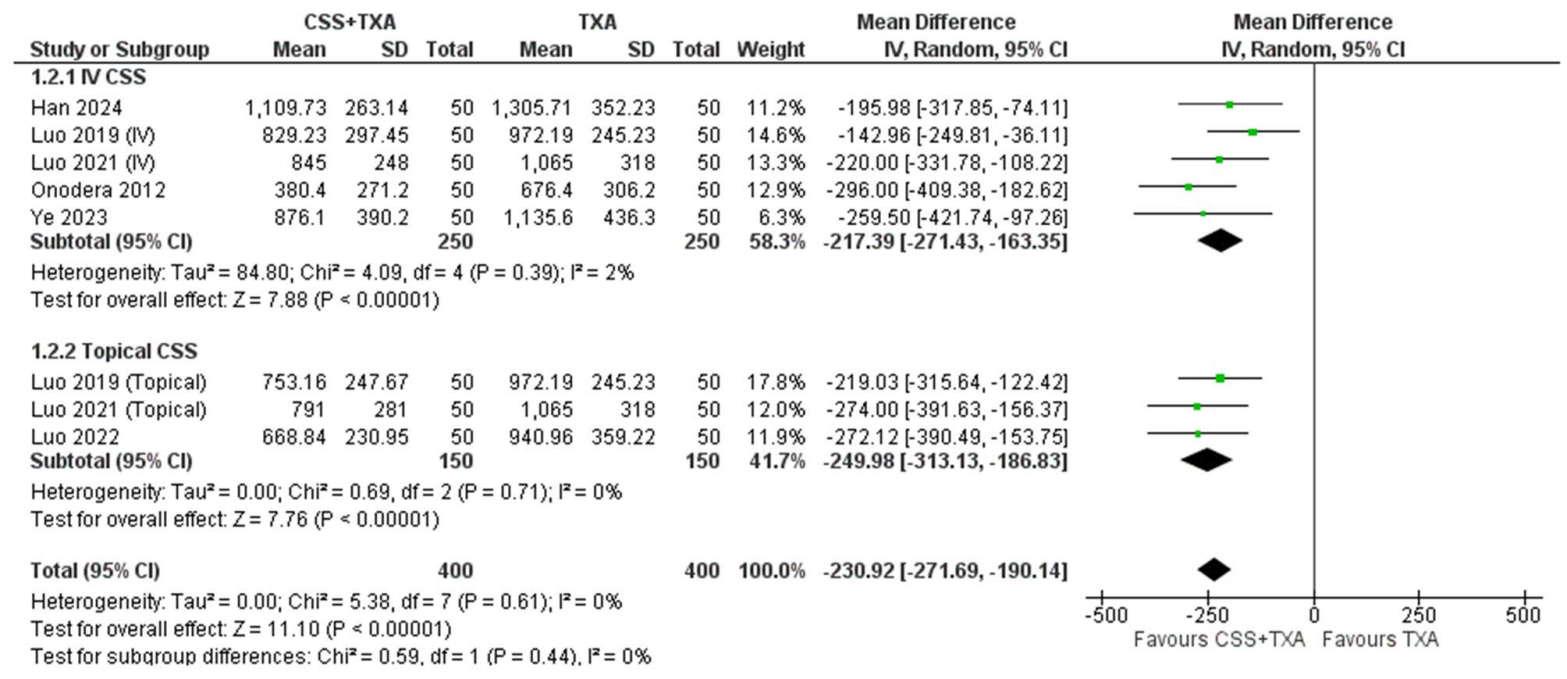
Shows the Forest plot of Total blood loss (TBL)

Subgroup analysis according to route of administration showed that both IV and Topical demonstrated significant reduction of TBL with Topical administration showing higher reduction in TBL than IV (MD= -249.98 ml, 95% CI [-313.13 to -186.83]; *P* < 0.00001) and (MD= -217.39 ml, 95% CI [-271.43 to -163.35]; *P* < 0.00001) respectively. Figure 3 presents a forest plot summarizing the meta-analysis results for total blood loss (TBL) outcomes. Another subgroup according to type of surgery -THA and TKA. THA and TKA arthroplasty showed comparable results in TBL (MD= -242.81 ml, 95% CI [-297.95 to -187.68]; *P* < 0.00001) and (MD= -217.70 ml, 95% CI [-300.57 to -134.83]; *P* < 0.00001) respectively as illustrated in Figure S1 in supplementary material.

**2- Intraoperative blood loss**

**Fig. 4:**
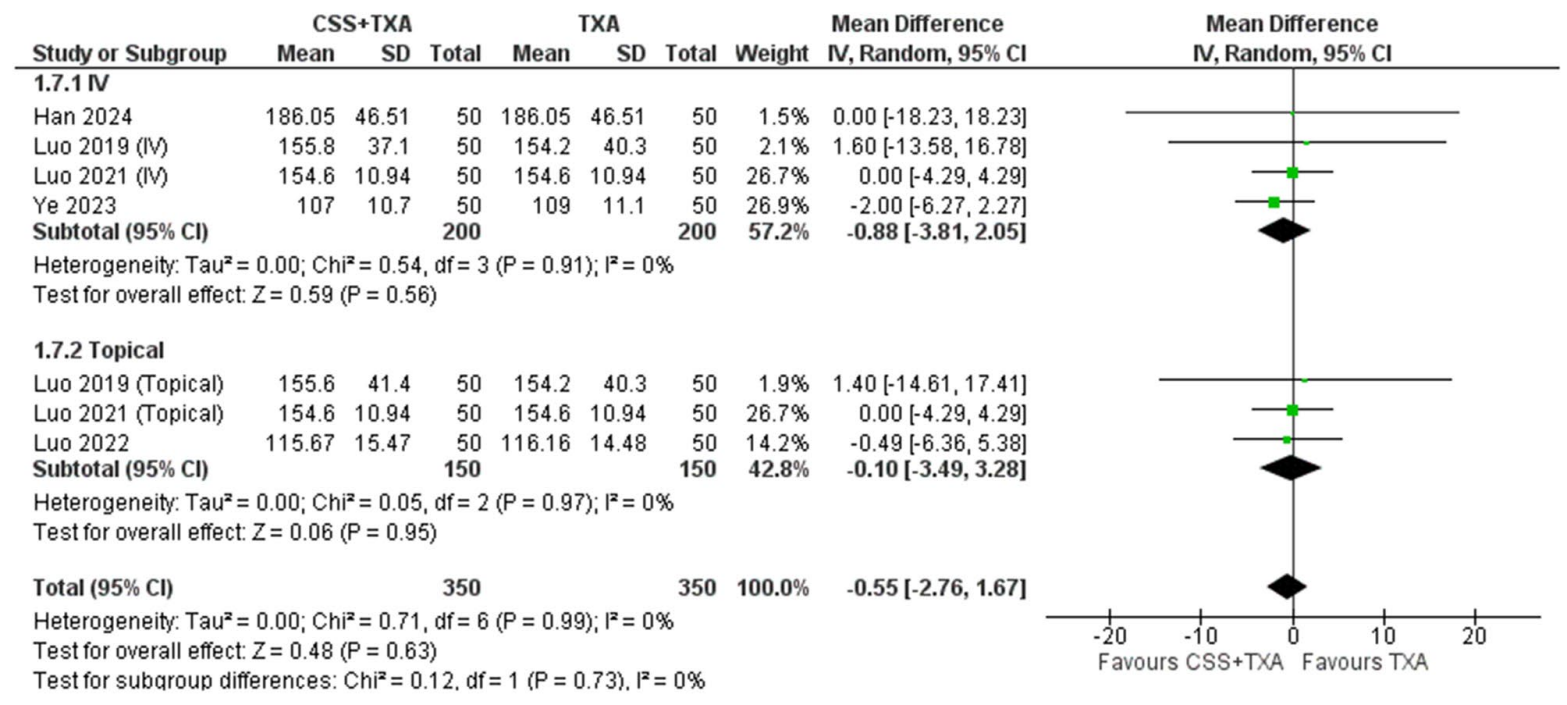
Shows the Forest plot of Intraoperative blood loss (IBL)

The meta-analysis of results of intraoperative blood loss indicates no statistically significant difference between TXA + CSS and TXA alone (MD= -0.55 ml, 95% CI [-2.76 to 1.67]; *p* = 0.63). Heterogeneity was minimal (τ² = 0.00; Chi² = 0.71, (*P* = 0.99); I² = 0%). Subgroup analysis revealed that neither intravenous (IV) nor topical administration of CSS combined with TXA resulted in a significant difference in intraoperative blood loss compared to TXA alone, with mean differences of (MD= -0.88 ml, 95% CI [-3.81 to 2.05]; *P* = 0.56) and (MD= -0.10, 95% CI [-3.49 to 3.28]; *P* = 0.97), respectively. Figure 4 illustrates the forest plot of intraoperative blood loss.

**3- Hidden blood loss**

**Fig. 5:**
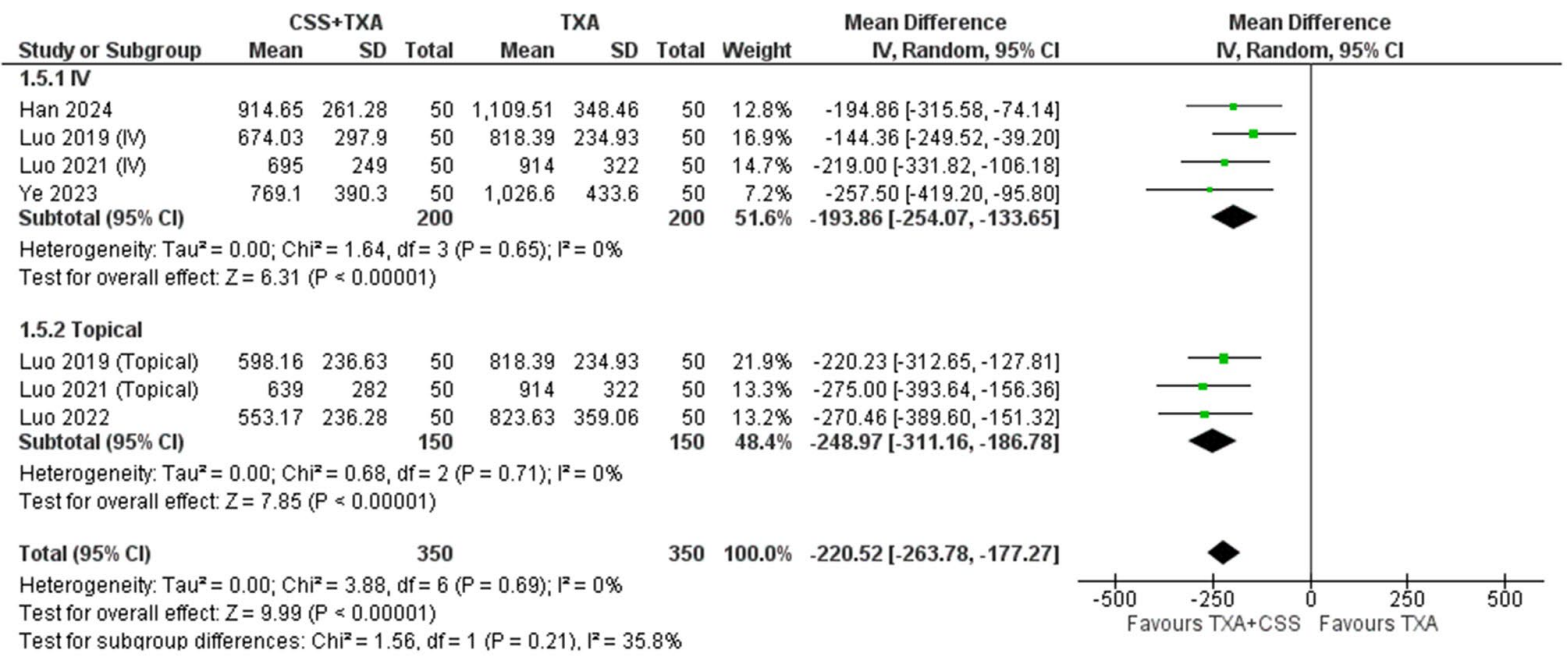
Shows the forest plot of Hidden blood loss (HBL)

A total of five studies (*n* = 700 participants) were included in the meta-analysis to assess hidden blood loss. The pooled effect estimate demonstrated that CSS + TXA was associated with higher reduction in hidden blood loss compared to TXA alone (MD= -220.52 ml, 95% CI [-26.78 to -177. 27]; *P* < 0.00001) with non-detected heterogeneity (*P* = 0.69), I2 = 0%). Subgroup analysis by route of administration revealed a more pronounced reduction with topical application (MD= -248.97 ml, 95% CI [-311.16 to -186.78]; *P* < 0.00001) than with intravenous administration (MD= -193.86 mL, 95% CI [-254.07 to -133.65]; *p* < 0.00001). Heterogeneity was minimal in IV ((*P* = 0.65); I² = 0%) and Topical groups ((*P* = 0.71); I² = 0%). Figure 5 shows the forest plot of hidden blood loss.

**4- Hemoglobin reduction**

**Fig. 6:**
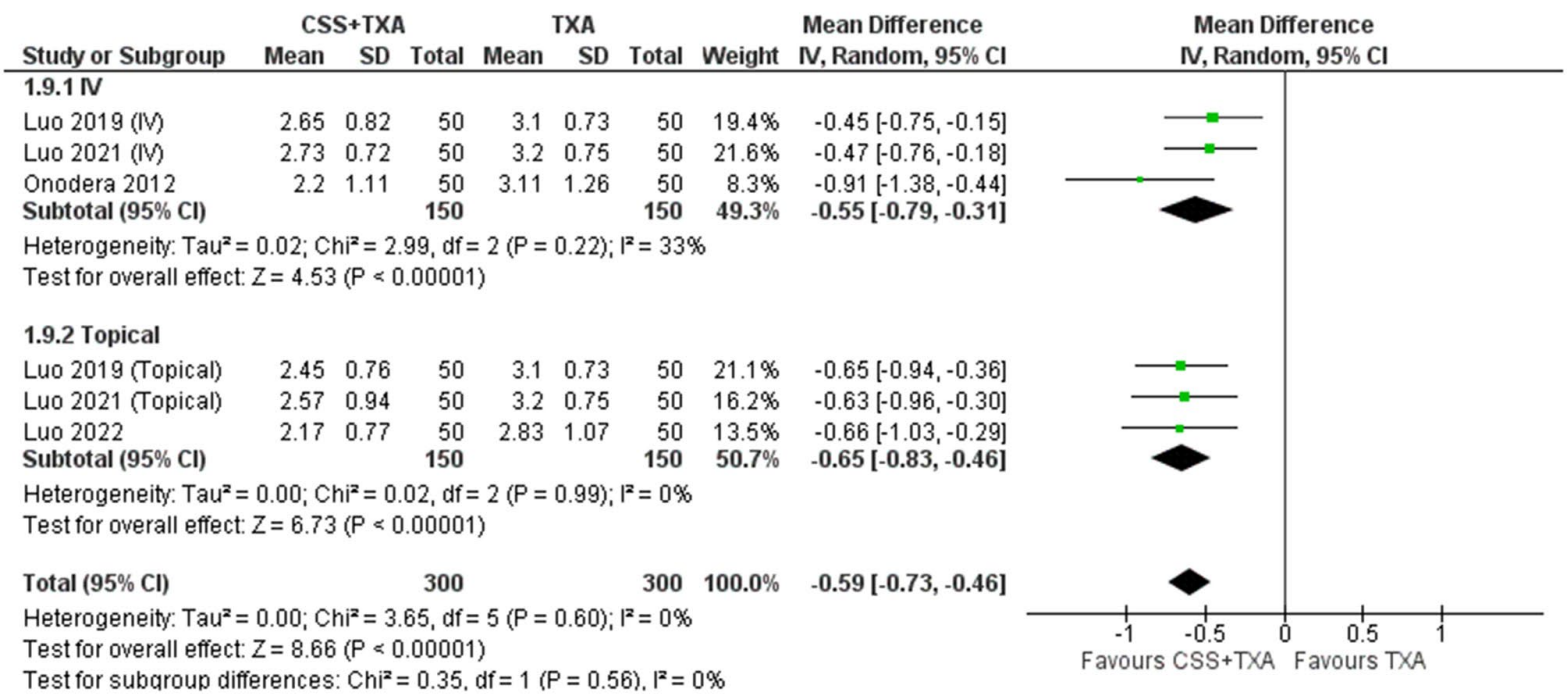
Shows the forest plot of Hemoglobin reduction

Meta-analysis demonstrated that CSS + TXA have shown less reduction of HB post operatively than TXA alone (MD= -0.59 mg/dl, 95% CI [-0.73 to -0.46]; *P* < 0.0001) with no heterogeneity detected ((*P* = 0.60); I² = 0%). Subgrouping according to route of administration showed that Topical administration had higher preservation of Hb (MD= -0.65 mg/dl, 95% CI [-0.73 to -0.46]; *P* < 0.00001) compared to IV (MD= -0.55 mg/dl, 95% CI [-0.79 to -0.31]; *P* < 0.00001) with minimal heterogeneity in both subgroups. Figure 6 illustrates forest plot of this outcome.

**5- Need for blood transfusion**

**Fig. 7:**
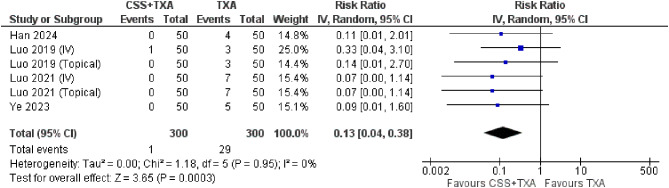
Shows the forest plot of Need for transfusion

The meta-analysis revealed that combination group showed less number of blood transfusion needed (RR = 0.13, 95% CI [0.04, 0.38], *p* = 0.0003) with minimal heterogeneity ((*P* = 0.95); I² = 0%) (Fig. 7).

### Secondary outcomes

#### Inflammatory markers

The meta-analysis showed persistent significant higher reduction in inflammatory markers; CRP ESR and IL-6 favoring CSS + TXA over TXA alone across three post operative endpoints at day one, day two and day thee. On day one, CRP showed a mean difference between both groups of MD = − 22.66 mg/dl (95% CI [-29.84, -15.47]: *P* < 0.00001), ESR by a MD= -11.71 mm/hr (95% CI [-18.77, -4.65]: *P* = 0.001) and IL-6 by a mean difference = -65.74 pg/ml (95% CI [-86.43, -45.06]: *P* < 0.00001). On day 2, CRP levels showed a further reduction with an MD of -35.84 mg/L (95% CI [-41.26 to -30.42]; *p* < 0.00001), ESR by -12.21 mm/h (95% CI [-15.90 to -8.52]; *p* < 0.00001), and IL-6 by -33.97 pg/mL (95% CI [-40.18 to -27.77]; *p* < 0.00001). By day 3, reductions were observed for CRP (MD= -31.89 mg/dl, 95% CI [-35.77 to -28.00]: *P* < 0.00001), ESR (MD= -16.83 mm/hr, 95% CI [-25.24 to -8.42]; *P* < 0.0001), and IL-6 (MD= -24.46, 95% CI [-28.47 to -20.44]; *P* < 0.00001). Heterogeneity analysis revealed various levels of inconsistency; non-significant heterogeneity were detected in CRP on day 2 (*P* = 0.29, I2 = 20%) and on day 3 (*P* = 0.10, I2 = 46%). Moderate heterogeneity was found on CRP day 3 (*P* = 0.29, I2 = 20%), ESR day 2 (*P* = 0.29, I2 = 20%), IL-6 day 2 (*P* = 0.29, I2 = 20%). Substantial heterogeneity was detected on CRP day 1 (*P* = 0.29, I2 = 20%), ESR day 1 (*P* = 0.29, I2 = 20%), ESR day 3 (*P* = 0.29, I2 = 20%), IL-6 day 1 (*P* = 0.29, I2 = 20%) and IL-6 day 3 (*P* = 0.29, I2 = 20%). Heterogeneity in ESR day 1 and 2 were solved by leave one out analysis. Figures S2–S10 in supplement illustrate forest plots of inflammatory markers outcomes.

#### Post operative pain

Post operative pain was assessed in three endpoints: one day, two days and three days.

- On day one post operatively, pooled analysis revealed a statically significant higher reduction in pain score in CSS + TXA compared to TXA alone (MD= -0.41 points, 95% CI [-0.62 to -0.21]; *p* < 0.0001). Substantial heterogeneity was observed ((*P* < 0.00001); I² = 90%) that could be solved by sensitivity analysis leaving out studies using NRS pain score -unlike other studies- Han 2024 and Ye 2023 ((*P* = 0.93); I² = 0%). (Figure S11 in supplement)
- On day two post operatively, meta-analysis showed lower pain score in CSS + TXA group compared to TXA alone (MD= -0.43 points, 95% CI [-0.69 to -0.18]; *p* = 0.001) with substantial heterogeneity ((*P* < 0.00001); I² = 96%). Heterogeneity was reduced by sensitivity analysis omitting NRS reporting studies to ((*P* = 0.08); I² = 51%). (Figure S12 in supplement)
- On day three post operatively, Pooled effect estimate showed persistent pain reduction in CSS + TXA group compared to TXA alone (MD= -0.29 points, 95% CI [-0.48 to -0.09]; *P* = 0.004) with substantial heterogeneity ((*P* < 0.00001); I² = 91%) that couldn’t be solved. (Figure S13 in supplement)

#### Operative time and length of hospital stay

Meta-analysis showed no statistically significant difference between combination of CSS + TXA and TXA alone in operative time or length of hospital stay (MD= -0.93 min, 95% CI [-2.70 to 0.84]; *p* = 0.30) and (MD = 0.03 days, 95% CI [-0.14 to 0.20]; *p* = 0.71) respectively. Minimal inconsistency were observed in operative time (I^2^ = 0%) and length of hospital stay (I^2^ = 0%). Forest plots are illustrated in Figures S14 and S15 in supplement.

### Safety outcomes

The meta-analysis of safety outcomes revealed that there was no statistically significant difference between CSS + TXA and TXA regarding wound complications or IM venous thrombosis (RR = 1.56, 95% CI [0.67 to 3.61]; *p* = 0.30) and (RR = 0.90, 95% CI [0.56 to 1.46]; *p* = 0.68) respectively. Figures S16–S17 in supplement illustrate forest plots of safety analysis.

### Leave one analysis

The leave-one-out sensitivity analysis was undertaken to evaluate the stability and sensitivity of the results obtained from the meta-analysis. In this analysis, studies were excluded one by one and the pooled effect estimates were recalculated to determine the influence of individual studies on the overall findings. In Post operative pain at day 3 outcome, by omitting Luo 2019 (IV group) or Luo 2019 (Topical group) or Luo 2022 at a once, results shifted to show non-significant difference indicating less robustness of findings of this outcome. However, leave one out analysis of the remaining outcomes showed no change of outcomes results indicating robust findings. Leave one out analysis leaving certain studies contributed to reducing heterogeneity in certain outcomes without affecting overall effect. Figures S19–S44 in supplement illustrate results of leave one out analysis.

## Discussion

By 2040, the number of total knee arthroplasty (TKA) performed is expected to be 1,222,988 surgeries while the number of total hip arthroplasty (THA) is projected to be 719,364 operations [43]. This significant increase is mostly attributed to the growing section of old population and the increasing incidence of degenerative joint diseases and traumatic injuries [43]. Despite the proven advantages of TJA in pain relief, joint function enhancement, and improving the overall quality of life for patients, this surgery technique still carries the weight of massive blood loss and the need for blood postoperative transfusion [44]. However, the use of TXA alone as a fibrinolytic medication has proven to significantly reduce the amount of blood loss and transfusion rates, some new evidence suggests that combining TXA with CSS can result in even greater reduction concerning blood loss, transfusion, and complications [45–47].

### Main findings

A systematic review and meta-analysis were conducted to assess the efficacy and safety of combined therapies using carbazochrome sodium sulfonate (CSS) with tranexamic acid (TXA) compared to the use of TXA alone in patients undergoing knee and hip arthroplasties. The results show that the coupled application of these two methods can yield great improvements in bleeding outcomes and postoperative recovery while maintaining a favorable safety profile.

#### Bleeding outcomes

The analysis showed each time that the combination use of CSS and TXA markedly lowered total and hidden blood loss relative to TXA only. This was observed irrespective of surgical technique employed (intravenous and topical CSS and knee and hip arthroplasty) and administration routes (topical or intravenous). The higher efficacy of the topical route highlights its potential as a preferred mode of delivery, offering targeted action at the surgical site, providing local control of the powerful anti-fibrinolytic agent. This is consistent with the mechanisms of both substances, since CSS has an additional capillary stabilizing action, thereby reducing microvascular bleeding caused by capillary damage [22].

The application of combination therapy was found to be applicable in both hip and knee arthroplasties suggests that its benefits are not solely surgery centered and can be generalized to other orthopedics surgeries. The intraoperative bleeding control had a small difference which could suggest that combination of CSS and TXA has its advantage over total and hidden blood loss. These findings could align with the CSS effect on capillary stabilization and microvascular bleeding. This understanding can assist clinicians devise tailored management techniques in patients at high risk of postoperative bleeding.

#### Preservation of postoperative Hemoglobin and transfusion requirements

The lower requirement of hemoglobin in the CSS + TXA group supports this depends on the combined regiment’s proper use suppression of both external and internal bleeding. Administering the medication topically, into and around joint capsule, instead of through an IV drip was more effective in preservation of hemoglobin levels which may be attributed to more localized hemostatic properties present in the topical dosages. This discovery has practical repercussions in reducing postoperative procedures like the aforementioned blood transfusions that were less common among the combination group and to some extent needed in the latter. Combined therapy has the potential to achieve less transfusion rates and, as a consequence, lower rates of transfusion complications, which may improve outcomes.

#### Inflammatory and Pain markers

The reduction of the inflammatory markers such as the CRP, ESR, and IL-6 with the CSS + TXA combination is significant which further emphasizes its potential anti-inflammatory benefits that extend beyond the scope of hemostasis, or it could be attributed indirectly to more hemostatic effect provided [48]. The postoperative inflammatory response when CSS combined with TXA is less, which implies that a better recovery is achieved due to light systemic inflammation and lesser tissues damages which supports faster functional rehabilitation [49]. These findings suggest that the combined treatment can help with hemostasis and enhance the recovery period, thereby becoming more comprehensive for the patients in the perioperative phase. The CSS + TXA combination post operative pain scores were more favorable at multiple time points, especially during the first few days following surgery. One possible reason for the reduction in pain may be indirectly due to the combined approach’s effect in reducing bleeding and inflammation, which then contributes to pain within the postoperative period. The marked presence of significant heterogeneity with some of the pain outcomes even after sensitivity analyses suggests variations within the study methodology and/or with the subjects. Indeed, this within subject variance indicates that further studies are needed to understand the interplay of factors that cause pain and to determine the patients who stand to gain maximum benefit from the combination therapy.

#### Safety and operative Efficiency

The safety analysis did not illustrate any significant disparities in wound complications or venous thromboembolism between the combination and TXA alone groups which suggest that the addition of CSS might not increase the risk for adverse events. The increased safety profile of the combination therapy is also proven by the lower transfusion rates. Moreover, the lack of marked differences in operative time and length of hospital stay indicates that the combined therapy, while effective in reducing blood loss, does not have a direct impact on these parameters. This could be due to the existence of other variables, including, but not limited, to differences in the surgical approach taken, the presence of medical conditions in the patient, or the threshold differences between systems which certainly seem to have more weight in defining how long the operation takes and how long the patient is hospitalized.

#### Stability and robustness of findings

The leave-one-out sensitivity analysis confirmed the robustness of the majority of primary and secondary outcomes quantitatively, largely implying that no single study had an overwhelming impact on the results. Significant outcomes of interest, in particular, total blood loss, hidden blood loss and hemoglobin preservation were not altered in their estimates even after multiple iterations illustrating the entrenched nature of the findings. For some outcomes like pain reduction on day 3 after surgery, there was a change that was statistically significant, but the combined effect of some studies now made it non-significant. These design aspects of the combined studies suggested that pain-reducing features of combined therapy did not persist into the later postoperative period starting from day three. This could have occurred due to other reasons like the body’s natural healing process or because of other influences of pain. While the overall findings are robust, this change of sensitivity within certain ranges of parameters poses problems of interpretation of the pain-related outcomes outside the early postoperative period.

### Clinical implications and future directions

The combination of CSS and TXA has a worth noticing clinical implication, CSS + TXA can significantly improve clinical outcomes for patients undergoing hip and knee arthroplasty. These therapies reduce both gross blood loss and hidden blood loss without compromising hemoglobin levels. Lowering the risk of blood transfusions in these patients enhances their overall safety and minimizes the risk of blood transfusion-related adverse events. Furthermore, compared to TXA alone, CSS + TXA may have a broader role in enhancing recovery and improving patient outcomes with its additional anti-inflammatory properties and decreased early postoperative pain. The enhanced efficacy associated with the CSS and TXA combination observed with topical administration makes this practical particularly in surgeries where there is a risk of excessive bleeding.

However, the lack of important changes regarding operative time and the length of hospitalization suggest that these outcomes are mediated by other factors such as the surgical approaches employed and organizational guidelines. In the future it would be important to confirm these results, improve dosing and administrational approaches, and determine long-term recovery effects. Furthermore, an analysis of the cost efficiency and practicability of the introduction of CSS + TXA into routine clinical practice will ensure its implementation.

### Previous research

As of today, no systematic reviews or meta-analyses have been published on the combined effect of carbazochrome sodium sulfonate (CSS) and tranexamic acid (TXA) on surgical bleeding. Likewise, no systematic reviews have been done on the CSS monotherapy relational efficacy. The majority of available literature consists of isolated studies that focused on the hemostatic effect of CSS within different surgical settings [50, 51]. Our study is the first systematic review and meta-analysis which aims to quantify the efficacy of combined CSS and TXA against TXA alone in reducing blood loss after arthroplasty and other surgical interventions.

### Strengths and limitations

There are strengths of the study which we have outlined. To begin, this is the first systematic review and meta-analysis which aims to evaluate the efficacy of combined usage of carbazochrome sodium sulfonate (CSS) and tranexamic acid (TXA), and there is no such study in the literature. We present a detailed and evidence-based evaluation of this combination therapy, supporting the clinicians faced with this difficulty. Additionally, our study maintains high standards of research methodology such as thorough search, inclusion criteria, and use of proper statistical methods to obtain robust results. Our study is also focused on both knee and hip arthroplasty, creating relevancy with the surgical disciplines and making the integration more stronger than weaker. When interpreting our findings, several limitations must be considered. One key limitation is the insufficient number of studies reporting range of motion outcomes, preventing pooled analysis. Although there were differences in sample sizes, CSS administration methods and doses, timing of drug delivery, surgical materials, and surgeon techniques, heterogeneity was minimal or absent. These variations improve the generalizability and external validity of the findings but may also introduce biases that could affect the pooled results.

### Conclusion

This meta-analysis confirms that the combination of carbazochrome sodium sulfonate (CSS) and tranexamic acid (TXA) is effective in minimizing total and hidden blood loss, maintaining hemoglobin concentrations, and lowering the necessity of transfusions in patients undergoing hip and knee arthroplasty. This treatment reduces inflammation and early postoperative pain. Moreover, the pain reduction is accentuated when the medication is applied topically rather than administered systemically. The therapy is not associated with increased risk of complications such as wound complications or venous thromboembolism; therefore, it has a good safety index. These findings should be confirmed in future studies where, in addition to the postoperative outcomes, the long-term effects of the procedure and its cost-effectiveness are assessed.

## Electronic supplementary material

Below is the link to the electronic supplementary material.

Supplementary Material 1

## Data Availability

Data is provided within the manuscript or supplementary information file.
