# Supplementary material for: Optimizing blood management in arthroplasty: a meta-analysis of carbazochrome sodium sulfonate and Tranexamic acid combination
Source: J Orthop Surg Res. 2025 Jul 17;20:668. doi: 10.1186/s13018-025-06038-x (PMC12273061; doi:10.1186/s13018-025-06038-x)
Supplement: Supplementary file 1 — Supplementary Material 1 [file 13018_2025_6038_MOESM1_ESM.docx]

**Supplementary materials**

**Title:**

**Optimizing Blood Management in Arthroplasty: A Meta-Analysis of Carbazochrome Sodium Sulfonate and Tranexamic Acid Combination**


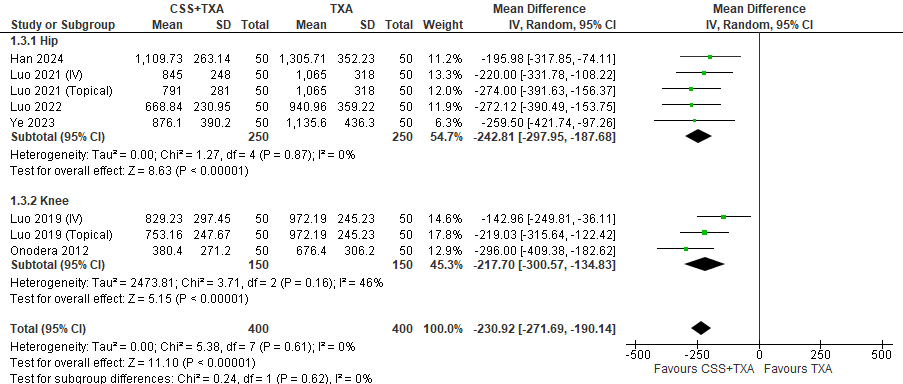


Figure S1 showing forest plot of Total blood loss subgroup according to type of surgery


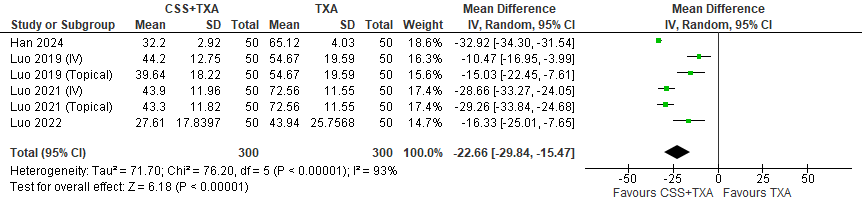


Figure S2 showing forest plot of CRP on day 1 PO


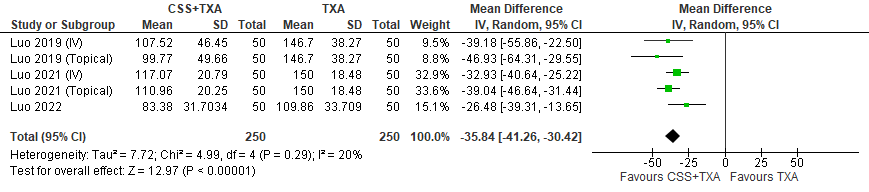


Figure S3 showing forest plot of CRP on day 2 PO


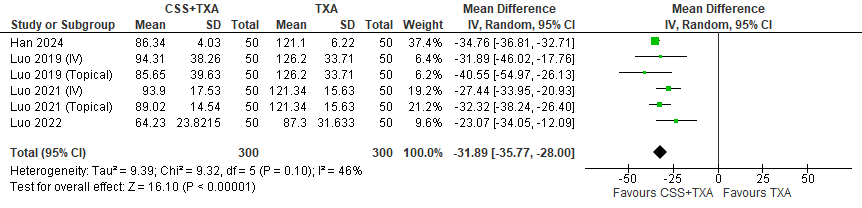


Figure S4 showing forest plot of CRP on day 3 PO

**A**

**
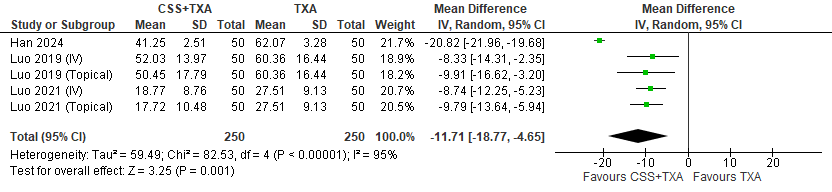
**

**B**

**
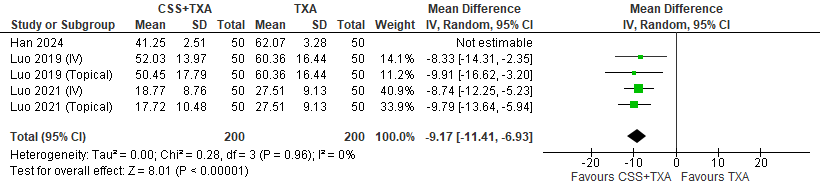
**

Figure S5 showing forest plot of ESR on day 1 PO, a: before sensitivity analysis, b: after sensitivity analysis

**A**

**
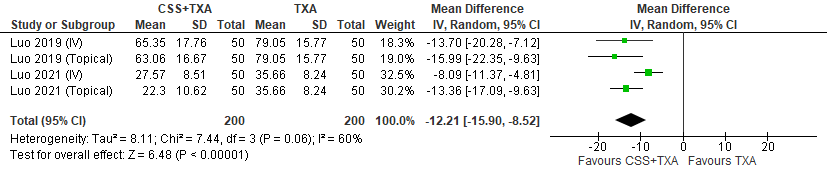
**

**B**

**
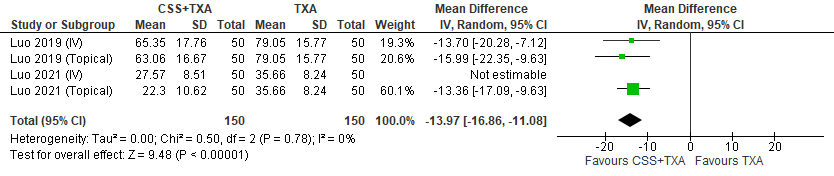
**

Figure S6 showing forest plot of ESR on day 2 PO, a: before sensitivity analysis, b: after sensitivity analysis


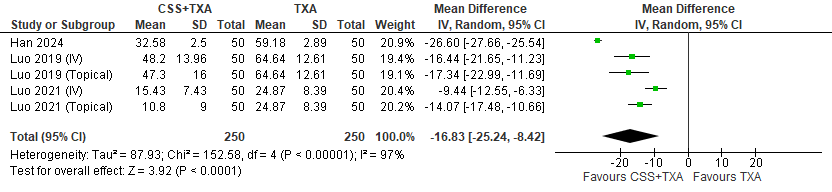


Figure S7 showing forest plot of ESR on day 3 PO


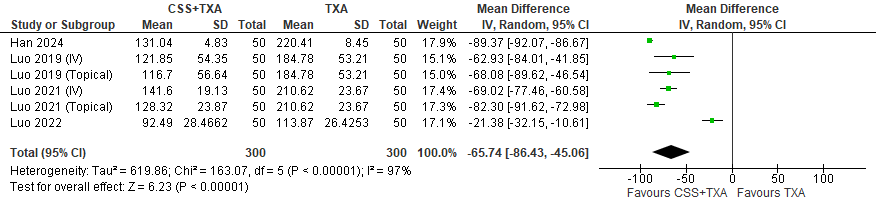


Figure S8 showing forest plot of IL-6 on day 1 PO


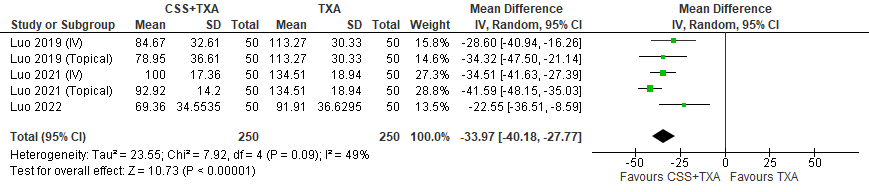

Figure S9 showing forest plot of IL-6 on day 2 PO


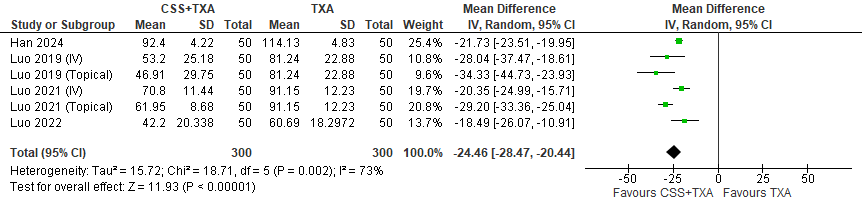

Figure S10 showing forest plot of IL-6 on day 3 PO

**A**

**
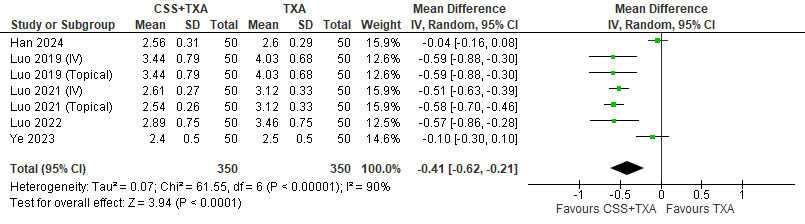
**

**B**

**
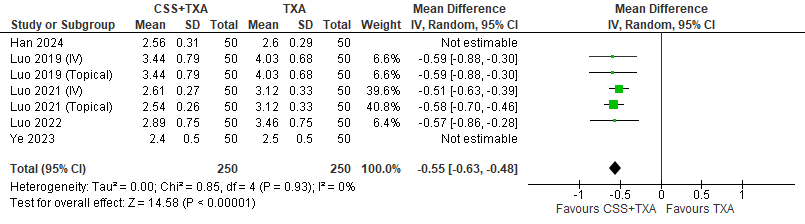
**

Figure S11 showing forest plot of Post operative pain on day 1 PO, a: before sensitivity analysis, b: after sensitivity analysis

\

**A**


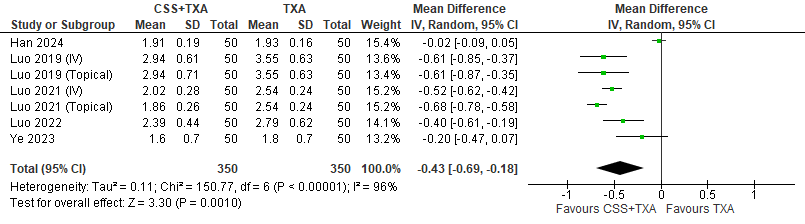


**B**


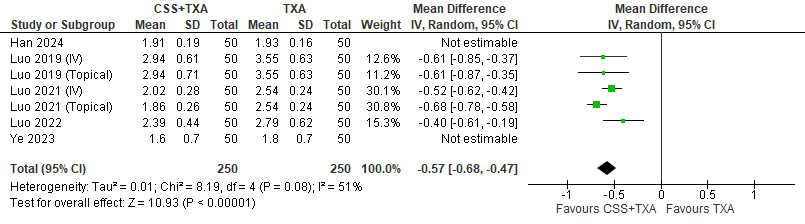


Figure S12 showing forest plot of Post operative pain on day 2 PO, a: before sensitivity analysis, b: after sensitivity analysis


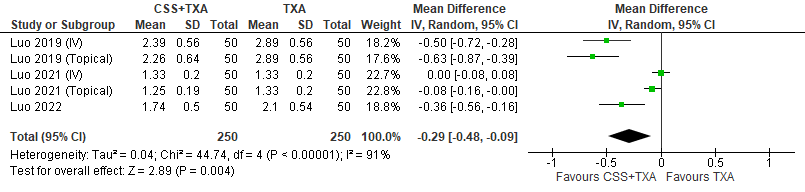


Figure S13 showing forest plot of Post operative pain on day 3 PO


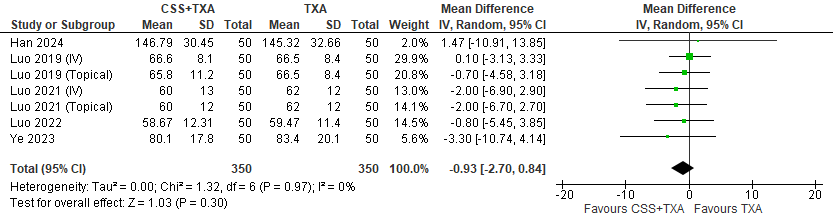


Figure S14 showing forest plot of Operative time (minutes)


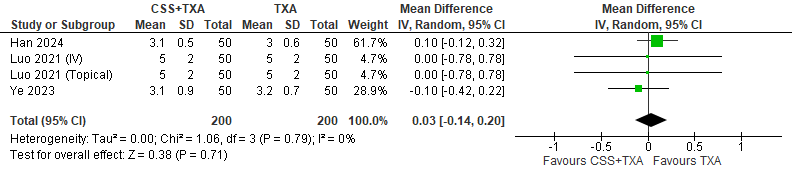


Figure S15 showing forest plot of Length of hospital stay (days)


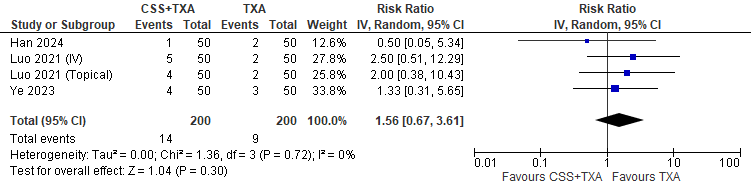


Figure S16 showing forest plot of Wound complications


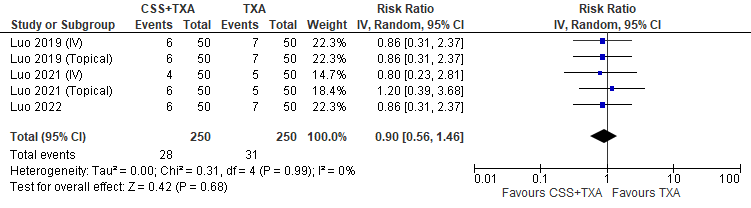


Figure S17 showing forest plot of IM venous thrombosis

**Leave-One-Out Analysis Figures**


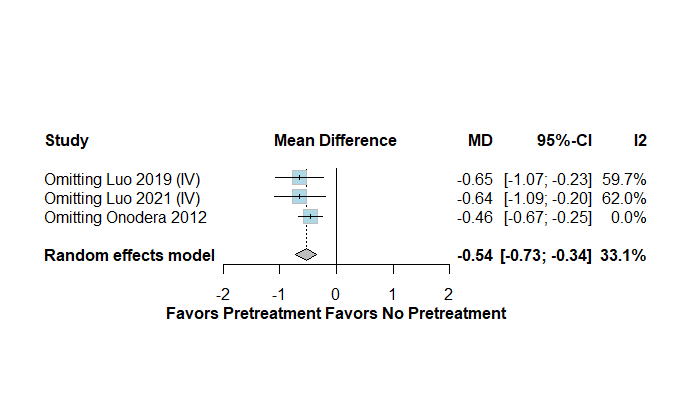


Figure S19 showing leave-one-out analysis of HB reduction IV


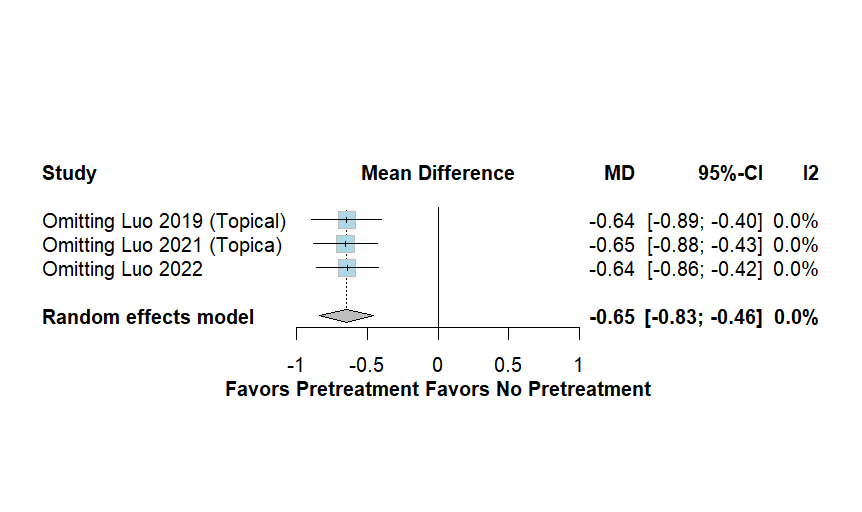


Figure S20 showing leave-one-out analysis of HB reduction Topical


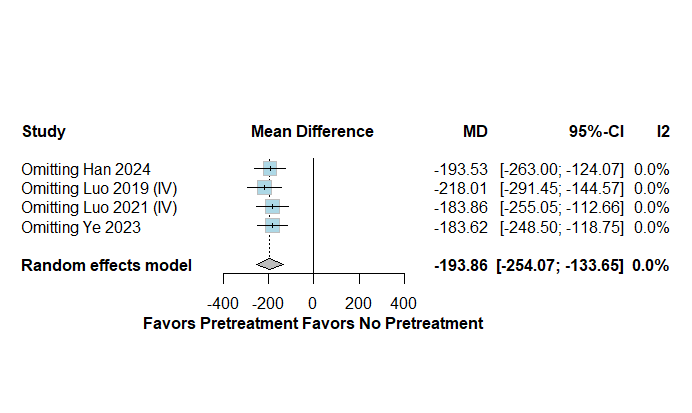


Figure S21 showing leave-one-out analysis of HBL IV


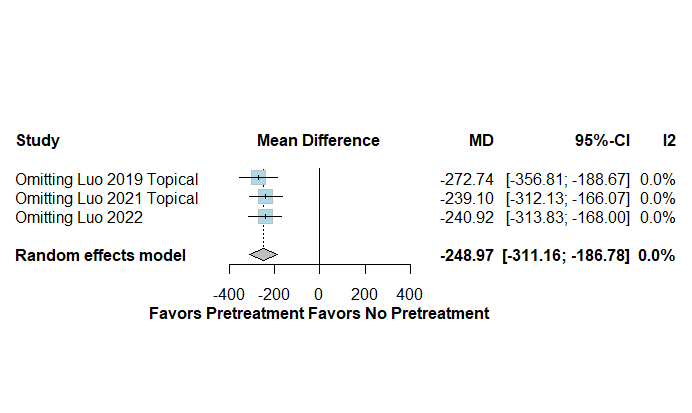


Figure S22 showing leave-one-out analysis of HBL Topical


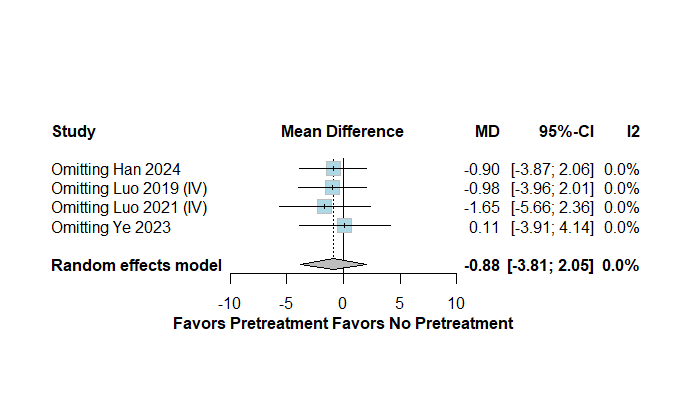


Figure S23 showing leave-one-out analysis of IBL IV


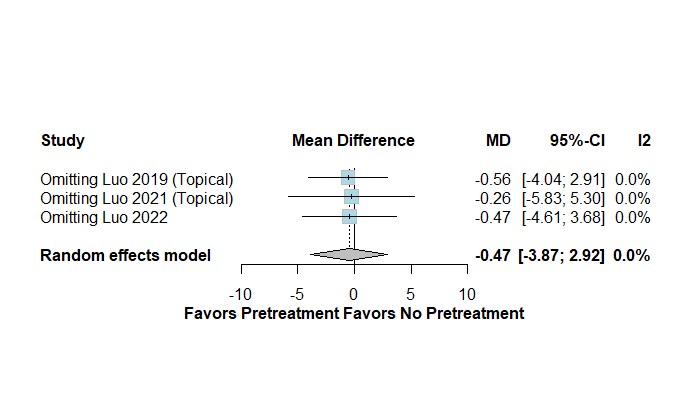


Figure S24 showing leave-one-out analysis of IBL Topical


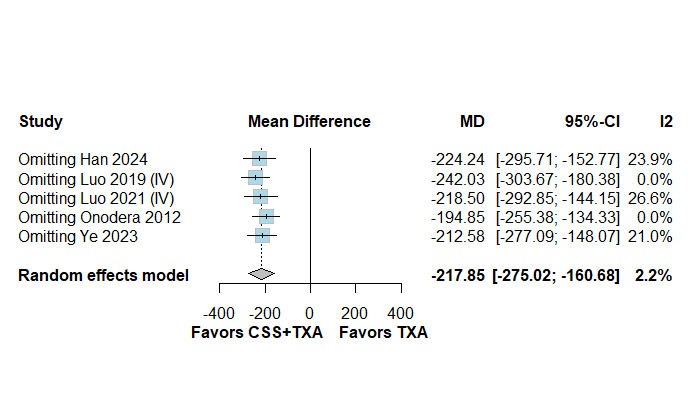


Figure S25 showing leave-one-out analysis of TBL IV


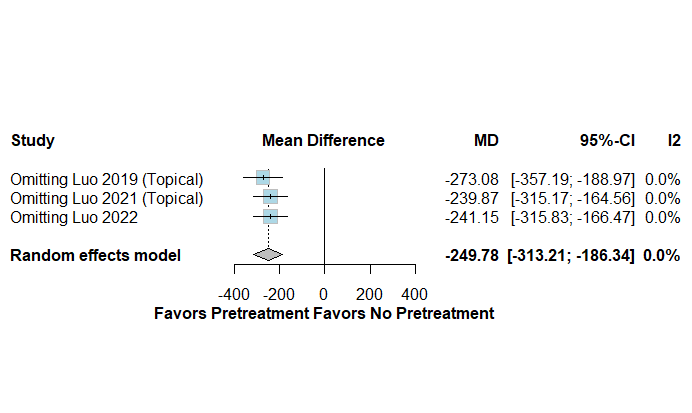


Figure S26 showing leave-one-out analysis of TBL Topical


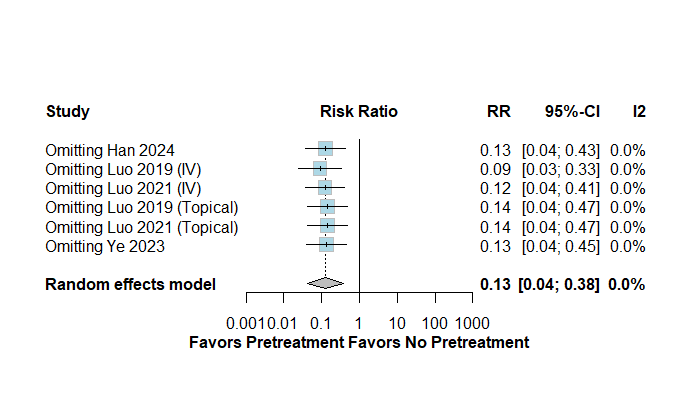


Figure S27 showing leave-one-out analysis of Blood Transfusion


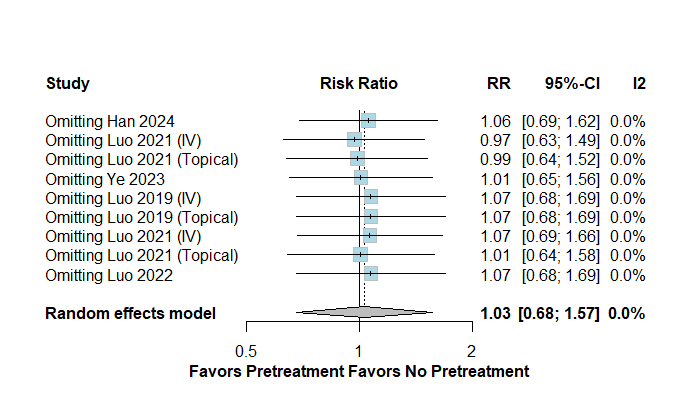


Figure S28 showing leave-one-out analysis of IM venous Thrombosis


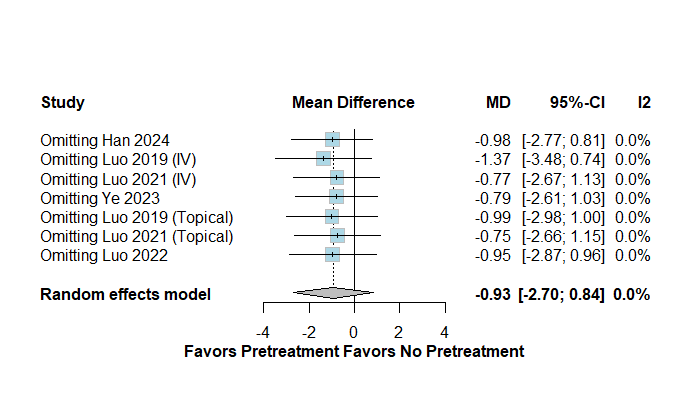


Figure S29 showing leave-one-out analysis of Operative Time


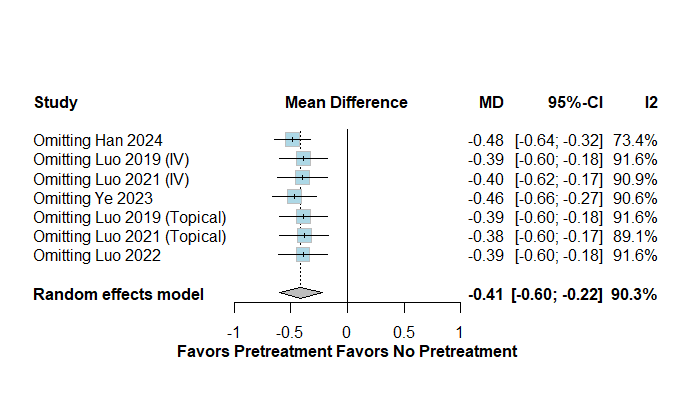


Figure S30 showing leave-one-out analysis of PO Pain Day 1


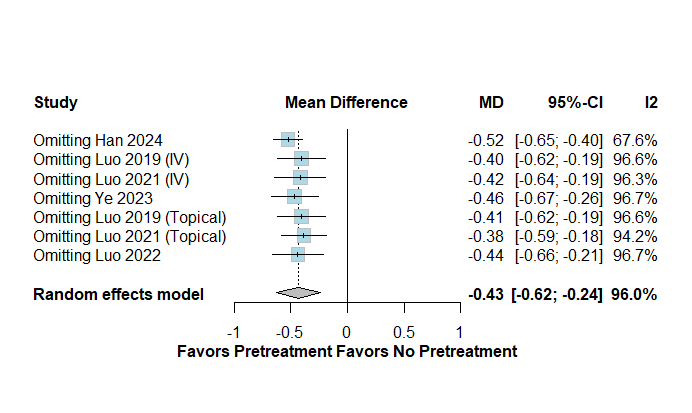


Figure S31 showing leave-one-out analysis of PO Pain Day 2


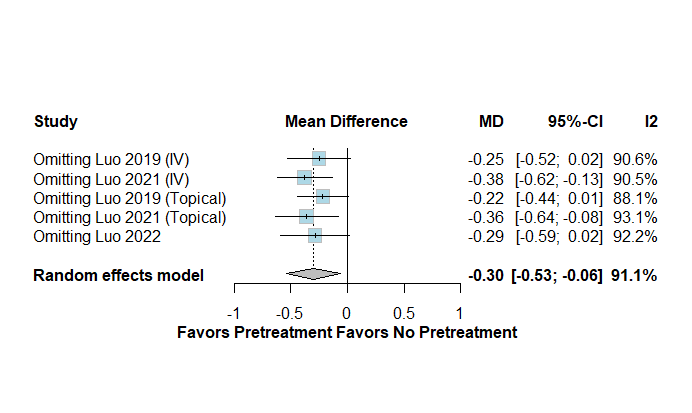


Figure S32 showing leave-one-out analysis of PO Pain Day 3


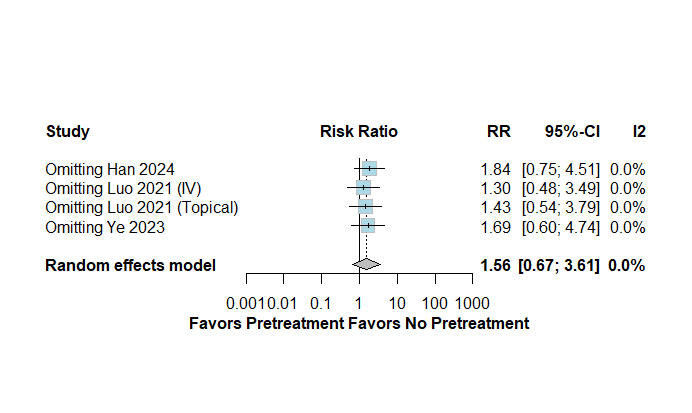


Figure S33 showing leave-one-out analysis of Wound Complication


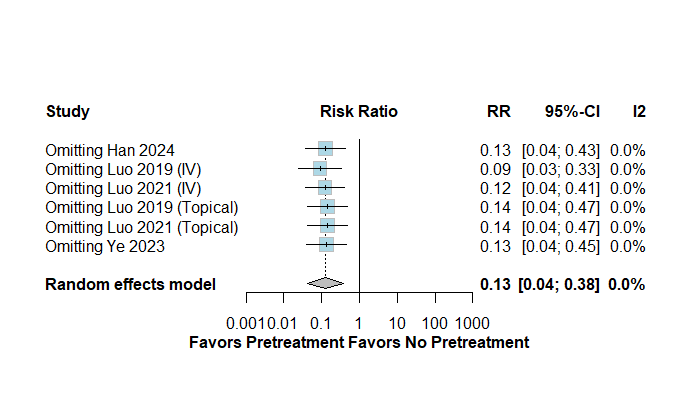


Figure S34 showing leave-one-out analysis of Blood Transfusion


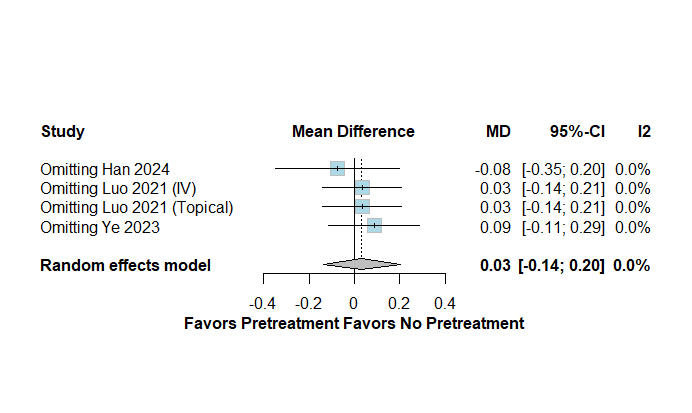


Figure S35 showing leave-one-out analysis of Hospital Stay


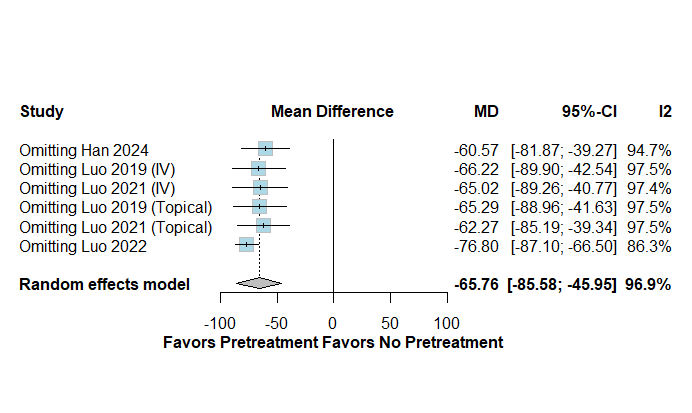


Figure S36 showing leave-one-out analysis of IL-6 (1)


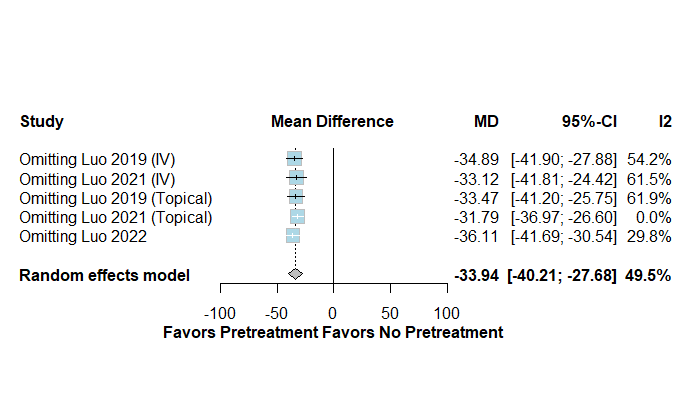


Figure S37 showing leave-one-out analysis of IL-6 (2)


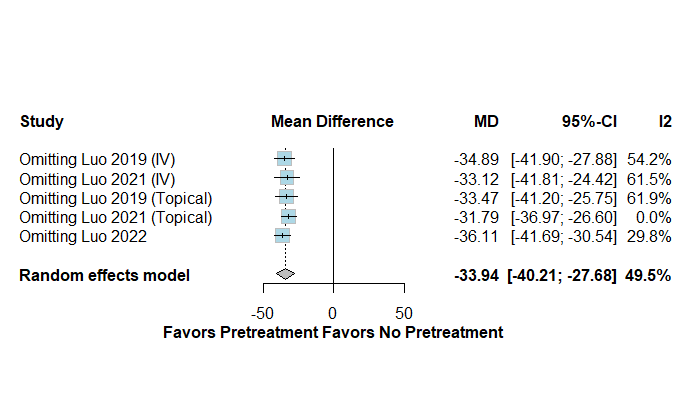


Figure S38 showing leave-one-out analysis of IL-6 (3)


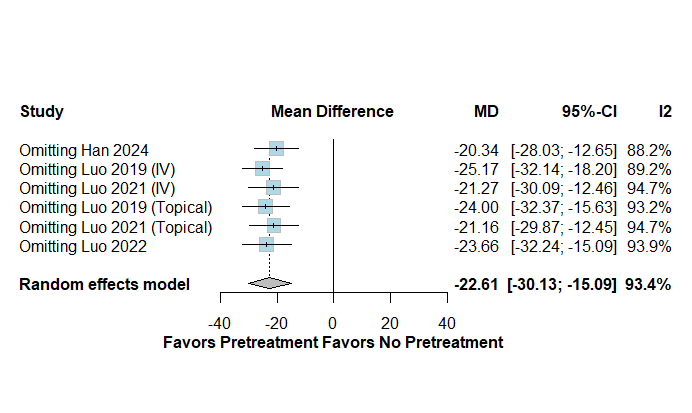


Figure S39 showing leave-one-out analysis of CRP (1)


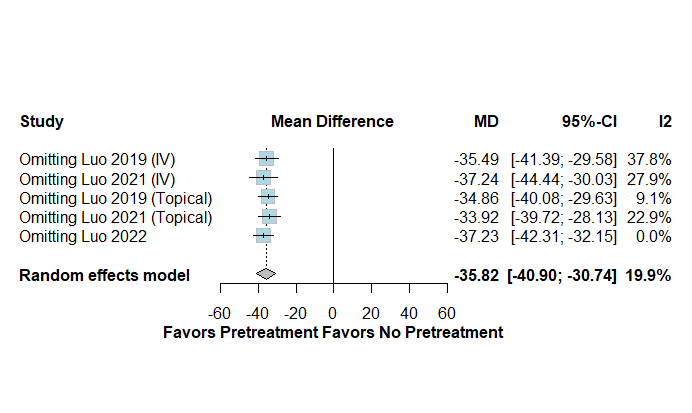


Figure S40 showing leave-one-out analysis of CRP (2)


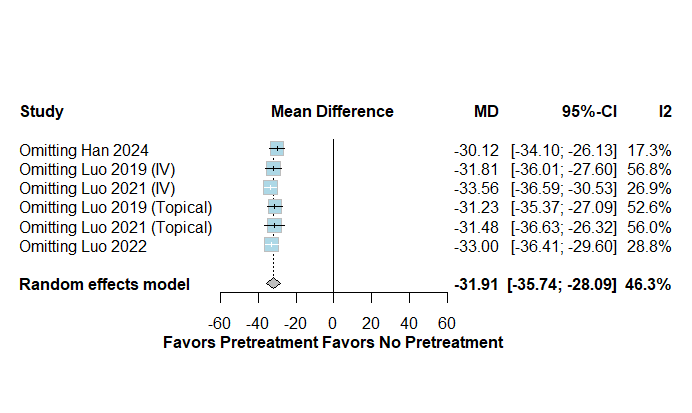


Figure S41 showing leave-one-out analysis of CRP (3)


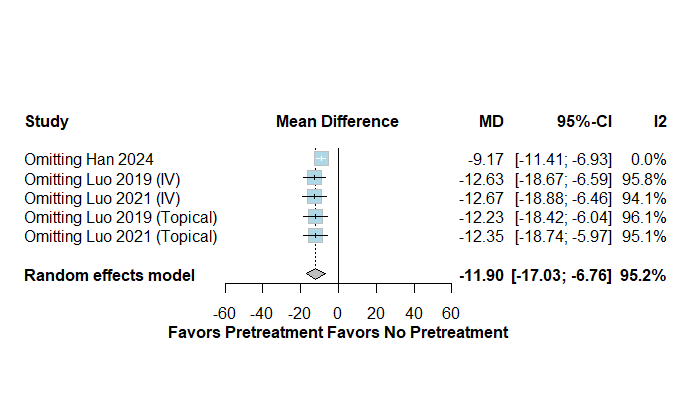


Figure S42 showing leave-one-out analysis of ESR (1)


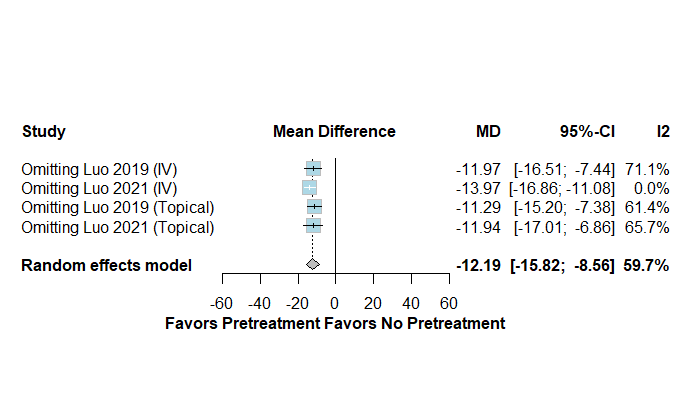


Figure S43 showing leave-one-out analysis of ESR (2)


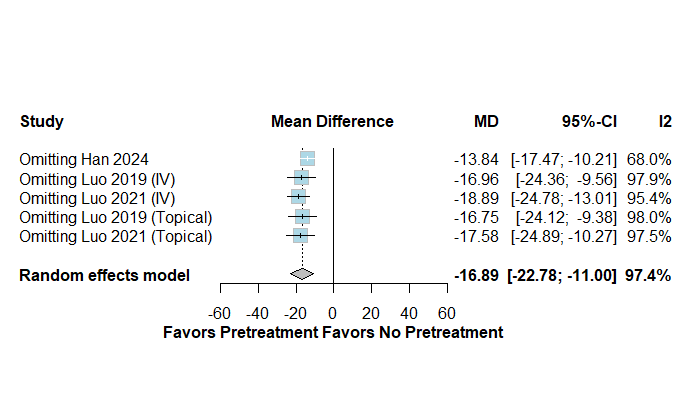


Figure S44 showing leave-one-out analysis of ESR (3)
